# Genetic association between *CDKN2B/CDKN2B-AS1* gene polymorphisms with primary glaucoma in a North Indian cohort: an original study and an updated meta-analysis

**DOI:** 10.1186/s12920-020-00855-1

**Published:** 2021-01-04

**Authors:** Nanamika Thakur, Manu Kupani, Rashim Mannan, Archna Pruthi, Sanjana Mehrotra

**Affiliations:** 1 Department of Human Genetics, Guru Nanak Dev University, Amritsar, Punjab India; 2 All India Institute of Medical Sciences, New Delhi, India

**Keywords:** *CDKN2B/CDKN2B-AS1*, Primary glaucoma, North Indian population, Genetic association study, *INK4* locus, SNPs

## Abstract

**Background:**

Variants in *CDKN2B*/*CDKN2B-AS1* have been reported to modulate glaucoma risk in several GWAS across different populations. *CDKN2B*/*CDKN2A* encodes tumor suppressor proteins p16^INK4A^/p15^INK4B^ which influences cell proliferation/senescence in RGCs, the degeneration of which is a risk factor for glaucoma. *CDKN2B-AS1* codes a long non-coding RNA in antisense direction and is involved in influencing nearby *CDKN2A/CDKN2B* via regulatory mechanisms.

**Methods:**

Current study investigated four SNPs (rs2157719, rs3217992, rs4977756, rs1063192) of aforementioned genes in a case–control study in a North Indian cohort. Genotyping was done with Taqman chemistry. In addition, an updated meta-analysis was performed.

**Results:**

Two SNPs, rs3217992 and rs2157719 were found to be significantly associated with the disease. The frequency of ‘T’ allele of rs3217992 was significantly lower in cases (POAG/PACG) [*p* = 0.045; OR = 0.80(CI = 0.65–0.99) and *p* = 0.024; OR = 0.73(CI = 0.55–0.96)], respectively than in controls. Genetic model analysis revealed that TT + CT genotype confers 0.73-fold protection against POAG [*p* = 0.047; OR = 0.73(CI = 0.54–0.99)] and trend assumed additive model gives 0.53 times higher protection against PACG progression. However the association of rs3217992 with POAG and PACG did not remain significant after Bonferroni correction. For rs2157719, the ‘C’ allele was found to be less prevalent among cases (POAG/PACG) with respect to controls. Cochran Armitage trend test assuming additive model revealed 0.77 and 0.64-fold protection against POAG and PACG respectively. Bonferroni correction (*p*_corr _= 0.003) was applied and the association of rs2157719 remained significant in PACG cases but not among POAG cases (*p* = 0.024). The ‘CC’ genotype also confers protection against primary glaucoma (POAG/PACG) among males and female subjects. The frequency rs1063192 and rs4977756 did not vary significantly among subjects, however the haplotype ‘CATA’ was found to be associated with increased glaucoma risk. An updated meta-analysis conducted on pooled studies on POAG cases and controls revealed significant association between rs1063192, rs2157719, rs4977756 and POAG except rs3217992.

**Conclusion:**

The study concludes significant association between *INK4* variants and primary glaucoma in the targeted North Indian Punjabi cohort. We believe that deep-sequencing of *INK4* locus may help in identifying novel variants modifying susceptibility to glaucoma. Functional studies can further delineate the role of *CDKN2B *and *CDKN2B-AS1* in primary glaucoma for therapeutic intervention.

## Background

Glaucoma, a group of optic neuropathies is an outcome of degenerating retinal ganglion cells (RGCs), resulting in optic disc cupping and visual loss [1]. It is the second leading cause of irreversible blindness worldwide [2]. The disease has a complex etiology and a wide clinical spectrum. Amongst different clinical forms, primary open-angle glaucoma (POAG) and primary angle closure glaucoma (PACG) are the most common. Multiple factors contribute to RGCs degeneration including elevated intra-ocular pressure (IOP), oxidative stress in retinal microenvironment and abnormal glial activation [3]. Linkage scans till date have identified 27 genetic loci which may harbor glaucoma related genes [4], yet there exists a wide gap between the heritability estimates in glaucoma and causative genes. Genome-wide association studies (GWAS) have been instrumental in bridging this gap and have led to the identification of several novel genetic loci which might affect genetic risk to POAG and PACG [5]. One such region is *INK4* locus at chromosome 9p21.3. Cyclin-dependent kinase inhibitor 2B (*CDKN2B*) and Cyclin-dependent kinase inhibitor 2A (*CDKN2A*) are two genes located adjacent to each other at *INK4* locus in a stretch of about 80 kb. They encode tumor suppressor proteins p16^INK4A^ and p15^INK4B^, respectively that inhibit cell cycle progression by forming complexes with cyclin-dependent kinase CDK4 or CDK6 [6]. *CDKN2A* also encodes for p14^ARF^ which is a splice-variant produced through an alternate reading frame [7]. Another gene located at *INK4* locus is *CDKN2B-AS1*, also known as *ANRIL* (antisense non-coding RNA), encodes a long non-coding RNA in the antisense direction and is involved in influencing the nearby *CDKN2A* and *CDKN2B* genes via regulatory mechanisms [8]. Genetic variants in three of the genes at *INK4* locus have been reported in several GWAS to be associated with glaucoma risk [8–10]. Initially thought to be associated with vertical cup disc ratio (VCDR) in a European cohort [11], and later on confirmed by Fan et al. [9], the association of *CDKN2B/CDKN2B-AS1* region with glaucoma was revalidated by several GWAS [8, 12–14]. Highly significant association of this region was observed with either the disease or its endophenotypes in many populations [8, 15–22] specifically with IOP. A study conducted by Gao and Jackobs in 2016, demonstrated increased vulnerability of RGCs in response to elevated IOP in mice homozygous for deletion in *INK4* locus stressing that the altered expression of these genes might modulate apoptosis of RGCs, eventually contributing to glaucomatous visual field defect [23]. The respective variants in these genes might also affect their levels in eye via miRNA mediated regulation as demonstrated by Ghanbari and co-workers [24]. Two 3′UTR variants (rs3217992 and rs1063192) of *CDKN2B* enhance affinity for the binding of miR-138-3p and miR-323-5p, respectively to *CDKN2B* and thereby affect miRNA mediated regulation of this gene [24]. Further strengthening the candidacy of *INK4* locus is the observation that p15^INK4B^ is a potential effector molecule for TGF-beta mediated cell-cycle arrest and thereby triggering axonal damage of optic nerve head (ONH) [6, 25]. Replication studies from Indian subcontinent have so far failed to detect significant association between *INK4* locus and glaucoma [26, 27], yet GWAS data from across the world provide a strong rationale to further validate the region. Therefore, in the present study, we evaluate the genetic association of four SNPs in *CDKN2B/CDKN2B-AS1* in a North Indian glaucomatous population.

## Methods

### Study participants

A hospital-based case–control association study was carried out; 461 unrelated cases with POAG and PACG were recruited along with 449 gender matched control individuals from Sardar Bahadur Sohan Singh Eye Hospital, Amritsar, Punjab after a complete ophthalmic examination. Approval for all research procedure was obtained from the Research Ethical Committee of Guru Nanak Dev University, Amritsar, India and the study protocols were according to the tenets of the Declaration of Helsinki. A written informed consent was obtained from all the study participants. A case was defined as having an open angle glaucoma if the patient had (1) an intraocular pressure (IOP) greater than 21 mm Hg in either of the eyes tested using Goldmann Applanation Tonometry and (2) glaucomatous optic nerve head damage defined as a vertical cup-disc ratio (CDR) of 0.7 or greater as adjudged clinically on slit lamp biomicroscopy using hand held + 90 D, this was confirmed using contrast enhanced fundus photograph on optical coherence tomography (OCT) as well as optic disc analysis or glaucomatous visual field defect as detected on Automated perimeter using Humphery’s Visual Field Analyser using Swedish Interactive Thresholding Algorithm (SITA) standard protocols. Individuals with known chronic systemic inflammatory, autoimmune or immunosuppressive disease as well as a pre-existing ocular disease (diabetic retinopathy, age-related macular degeneration) were excluded from the study. Individuals with history of corticosteroids, non-steroidal anti-inflammatory drugs, and topical use of steroids or prostaglandin analogues were also not recruited. Study controls, as examined by tonometry, slit lamp examination, CDR measurement and visual field assessment had normal IOP, optic disc and visual field. It was also ensured that the control samples did not have any family history of glaucoma.

### Sample collection, DNA isolation and genotyping

Venous blood was collected in EDTA vacutainers for genotyping experiments and stored at – 80 ºC till further use. Genomic DNA was extracted using standard phenol chloroform method [28]. Quantification of extracted DNA was done using NanoDrop ND-2000 spectrophotometer (NanoDrop Technologies, Wilmington, DE). Genotyping of *CDKN2B* 3′ UTR A > G (rs1063192), *CDKN2B* 3′ UTR C > T (rs3217992), *CDKN2B-AS1* intergenic T > C (rs2157719), *CDKN2B-AS1* intergenic A > G (rs4977756) was performed using predesigned TaqMan real time PCR genotyping assay (Applied Biosystem, Foster city, CA; Catalogue no. C_2618046_10, C_341975_10, C_2618013_10 and C_11841829_10 respectively). Reactions were carried out in 48 well plates, in a 10 µL reaction volume using genomic DNA, TaqMan genotyping master mix (2X) (Applied Biosystems) and TaqMan Genotyping Assay (40X). The allelic discrimination (AD) assay was performed using StepOne Real Time PCR System (Applied Biosystems, Foster city, CA). Genotypes were scored by StepOne software v2.3 and manually confirmed by looking at the amplification plots. As a quality control measure, genotypes of few samples were retested by Sanger sequencing. The accession ID of sequences from which the primers were designed are NM_004936.3 (rs1063192), NM_004936.4 (rs3217992), NC_000009.12 (rs2157719 and rs4977756).

### Statistical analysis

Descriptive statistical analysis for demographic and clinical characteristics of the study participants was performed using SPSS software. Student’s *t* test was used to compare the baseline data among cases and controls. The results are represented as mean ± standard deviation (SD). Hardy Weinberg Equilibrium (HWE) and genetic association analysis was performed using PLINK software (v1.07). The distributions of genotype and allele frequencies among cases and controls were assessed by using 3 × 2 and 2 × 2 chi-square contingency tables and overall effect was determined in the form of Odd’s ratio (OR) under different genetic models; dominant, recessive, co-dominant and additive at 95% confidence interval (CI). Haplotype software was used to determine haplotype frequencies. Pairwise linkage disequilibrium (LD) for the selected gene variants was calculated using the Expectation–Maximization (EM) algorithm as implemented in PLINK software. *p* values of less than 0.05 were considered to be statistically significant. Bonferroni correction was applied to reduce type I error (4 SNPs × 4 models); thus, *p* value was set at 0.05/16 = 0.003 for genetic model analysis.

### Meta-analysis

#### Literature survey strategy

In the present study, a comprehensive literature survey was also performed, to find all the studies investigating the association among *CDKN2B/CDKN2B-AS1* gene polymorphisms (rs3217992, rs1063192, rs2157719 and rs4977756), with primary open angle glaucoma. Online search using some popular websites including “Pubmed”, “Medline”, “Google scholar” and “Embase” was conducted upto 20-08-2019. The following keywords were used in the search: Glaucoma, POAG, *CDKN2A/CDKN2B*, rs3217992, rs1063192, rs2157719 and rs4977756 and glaucoma, ocular hypertension and glaucoma. While searching literature, articles published only in English language were included in the study. Reference lists of original articles were screened to include maximum number of papers for the present meta-analysis.

#### Inclusion/exclusion criteria for meta-analysis

**Fig. 1:**
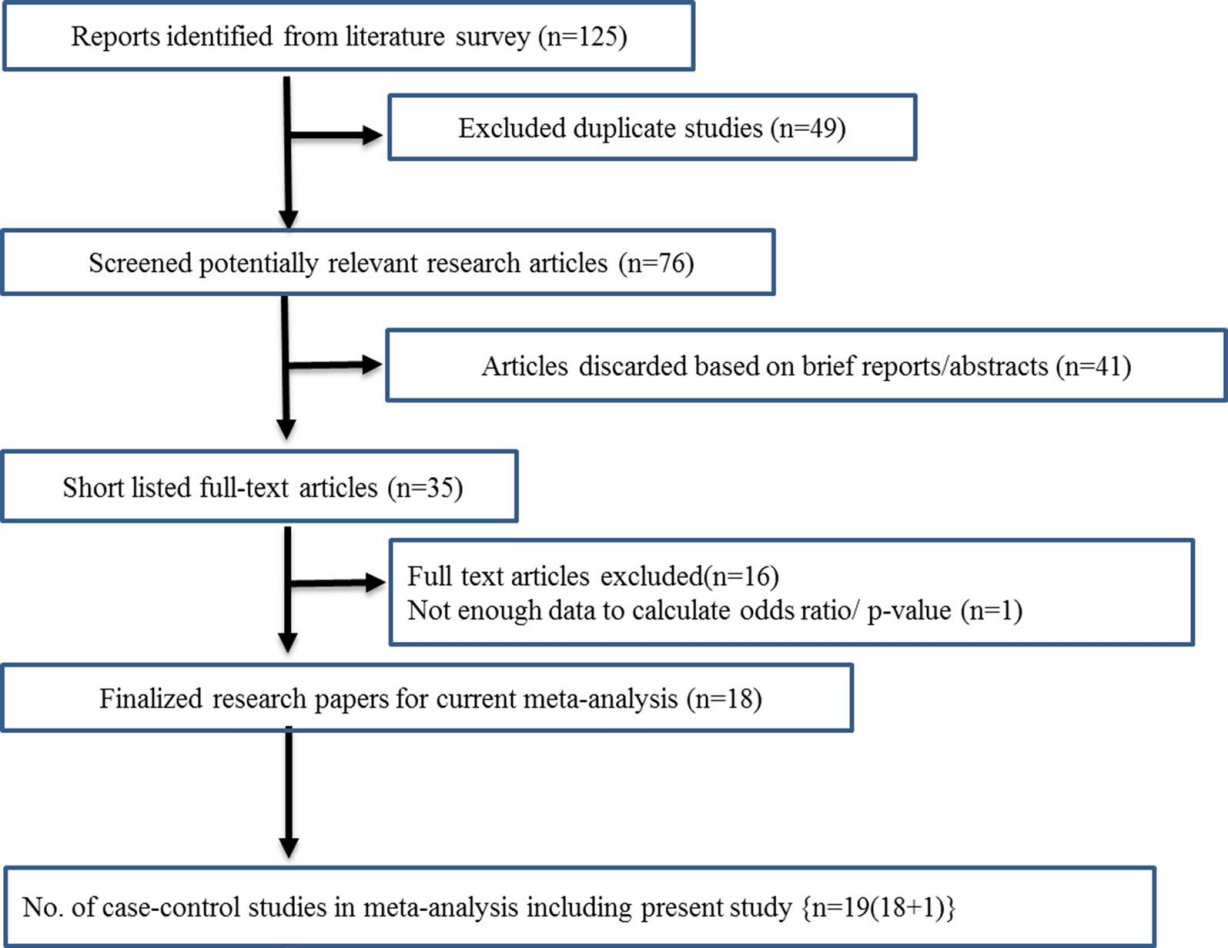
Flow chart depicted the outlining of selection procedure for the inclusion of the studies in the systemic review and meta-analysis

Only case–control studies conducted to examine the association between POAG and *INK4* genetic variants were included. The inclusion of the case–control subjects in these studies was done according to the standard diagnosis criteria of glaucoma. Reported odds ratio (OR) or relative risk (RR) estimates with 95% confidence interval (CI), raw *p* values were incorporated in the present meta-analysis. Studies on secondary glaucoma or other types of primary glaucoma (PCG, PACG) and those for which odds ratio could not be calculated were excluded. Reviews, short/brief reports, abstracts which did not include details about genotypic frequencies, allelic frequencies, *p* values, odds ratios etc. were excluded from current analysis as shown in Fig. 1.

#### Data extraction and quality assessment

Two authors (Nanamika Thakur, Manu Kupani) extracted all the required and relevant information regarding the study design, first author, year of publication, place (country and ethnicity) where the study was conducted, definition of glaucoma, sample size, information about genotype number in both cases and controls, outcomes measured with 95% CI and *p* value adjusted variables. Assessment of the quality of each study was done by two authors using the Newcastle–Ottawa quality assessment scale (NOS) tool criteria [29]. NOS criteria comprises three parameters of quality assurance which include: selection (Case defined adequately, representativeness of cases, selection of controls and definition of controls), comparability (controls matched for age, gender and other confounders either at design stage or during analysis) and exposure (ascertainment of exposure, same selection method for ascertainment of cases/controls and same non response rate) [29]. The total score was either 7 or 8 which indicated that included studies had high-quality scores. The individual score for all the studies has been given as Additional file 1: Table S1 (The Newcastle-Ottawa Scale for the assessment of case-control studies included in the meta-analysis). Any kind of conflicts regarding quality assurance was resolved by group discussions.

#### Statistical analysis

Present meta-analysis was done by software R (Version 3.6.1). Statistical analyses were made on estimates obtained from case control studies. Pooled adjusted odds ratio (OR) and its 95% CI was used as a measure to assess the association between *CDKN2B-AS1* gene variants and glaucoma among cases and controls. Pooled OR was calculated for dominant model, heterozygous model, and recessive model. Since all the included studies were conducted on different ethnic groups with different sample sizes, therefore heterozygosity was tested using I^2^ statistic. In heterozygosity testing, *p* value was < 0.05 and I^2^ value was > 50% which indicated large difference among studied groups, hence data was combined using random effect models. The fixed effects model was used for studies with low heterogeneity. The *I*^2^ value of > 75%, < 75%, < 50%, and < 25% represent considerable, substantial, moderate, and low heterogeneity, respectively. Sensitivity analysis was also performed by eliminating single study at a time to assess whether a single study could affect the overall results. Publication bias was investigated manually by observing funnel plots visually. Normal distribution (scatter plots) of studies in funnel plot indicated no publication biasing. These results were further verified by using Begg and Egger’s regression tests that were applied only if the number of included studies in meta-analysis was   ≥ 10. *p* value < 0.05 was considered as statistically significant for the overall effect.

## Results

**Fig. 2:**
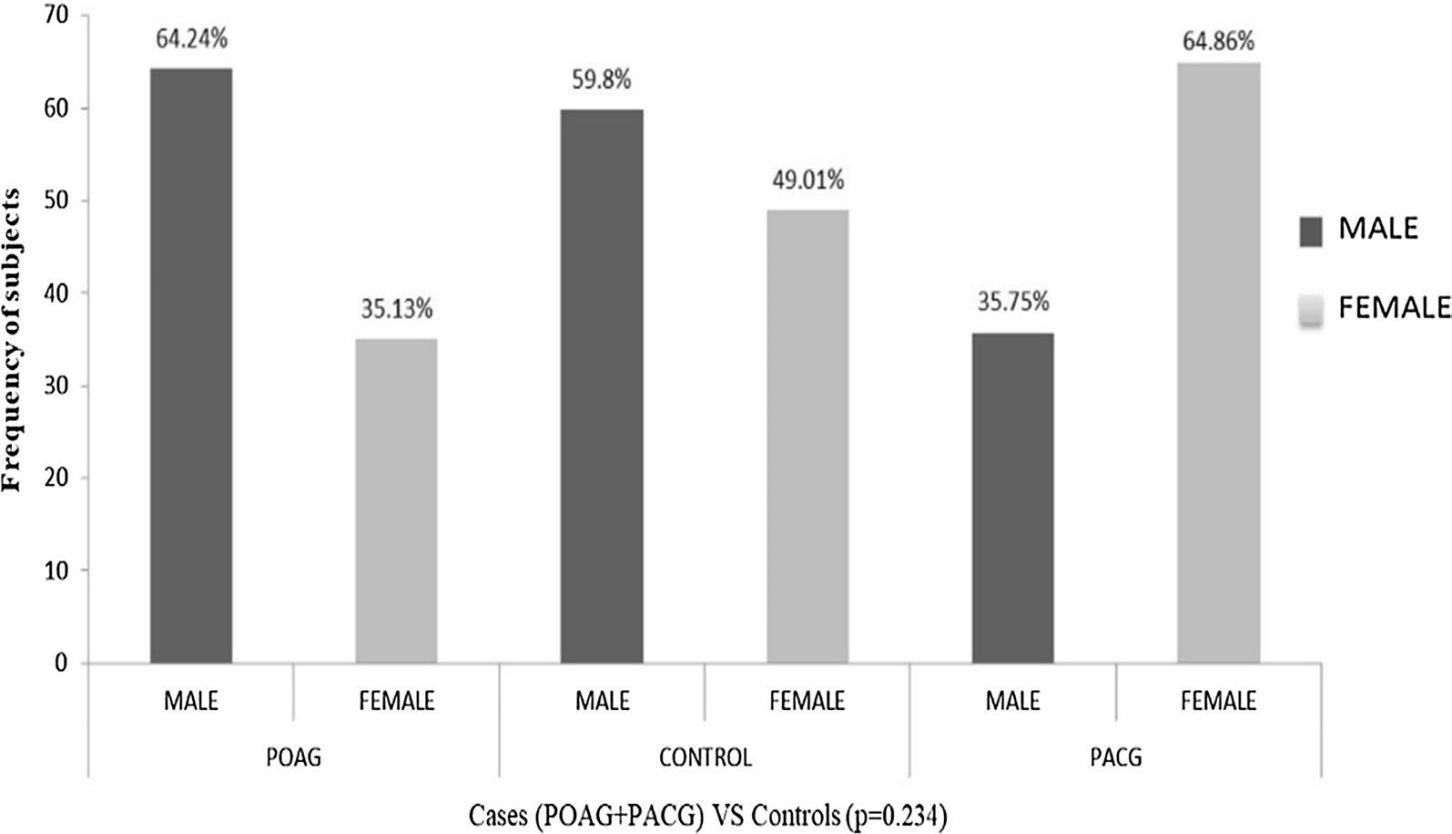
Frequency distribution of males and females among POAG and PACG cases with respect to control subjects

The association of *CDKN2B* (rs3217992, rs1063192) and *CDKN2B-AS1* (rs2157719, rs4977756) gene polymorphisms with primary glaucoma cases was investigated in a total of 910 samples: 449 controls having cataract, 313 POAG cases and 148 PACG cases. All cases and controls were age and sex matched. Demographic and clinical parameters of the study participants were compared using *t* test, values represented as mean ± standard deviation (SD) in (Table 1). The success rate of genotyping for four variants was different. The total sample size for each of the variant is mentioned in the respective result tables. Prevalence of POAG was more among males (64.24%) as compared to PACG cases (35.75%), whereas the frequency of PACG was more in females (64.86%) as compared to POAG (35.13%) as given in Fig. 2. Overall there was no significant difference in the number of males and females in both cases and controls (*p* = 0.234). The genotype counts in controls for three SNPs (rs3217992, rs1063192 and rs4977756) followed HWE frequencies but rs2157719 showed significant deviation in controls (*p* value = 0.0003). To rule out genotyping errors, the amplification plots were rescored and genotyping was repeated on randomly selected samples. No genotyping discrepancy was observed. Since the genotypes for other three SNPs in the present study were in accordance with HWE frequency in controls, it is highly unlikely that the deviation is due to selection or nonrandom mating. However, to avoid any false positive association, Cochran Armitage (CA) trend test assuming additive model was applied for analyzing rs2157719 in addition to dominant, recessive and codominant models. The advantage of applying CA test is that it does not assume HWE [30].

**Table 1:** Baseline demographic and clinical parameters among cases and controls

Factors	Cases (Mean ± SD)	Controls (Mean ± SD)	*p* value
Age	60 ± 12.61	58 ± 12.78	0.020
CD right eye	0.72 ± 0.39	0.23 ± 0.08	0.000*
CD left eye	0.73 ± 0.39	0.25 ± 0.09	0.000*
IOP right eye	22.41 ± 8.89	14.09 ± 3.51	0.000*
IOP left eye	22.90 ± 9.89	14.24 ± 3.43	0.000*

*p value < 0.01 (corrected *p* value) was considered to be statistically significant

### Frequency distribution of rs3217992 C > T and rs1063192 A > G 3′ UTR polymorphisms of *CDKN2B* among POAG and PACG cases with respect to control subjects

For genetic association analysis, data was stratified into two groups, POAG cases and PACG cases and comparison was done with controls. Genotype and allele frequencies of the SNPs among cases and controls are given in Table 2. The genotype counts for both the variants were found to be consistent with Hardy Weinberg frequencies. The frequency of minor allele (T) for rs3217992 was significantly lower in cases (POAG/PACG) [*p* = 0.045; OR = 0.80 (CI = 0.65–0.99)] and [*p* = 0.024; OR = 0.73 (CI = 0.55–0.96)] respectively than control subjects. The frequency of risk allele i.e. C allele of rs3217992 C > T polymorphism was found to be significantly higher in POAG (60.23%) and PACG cases (62.59%) as compared to controls (55.07%) as mentioned in Table 2. Further, genetic model analysis revealed that TT + CT genotype conferred 0.73 [*p* = 0.047; OR = 0.73(CI = 0.54–0.99)] and 0.69-fold [*p* = 0.061; OR = 0.69(CI = 0.46–1.01)] protection against POAG and PACG development and in additive model, TT genotype gave 0.53-fold protection against PACG progression (*p* = 0.03). However, the association did not remain significant after Bonferroni correction. The distribution of allele frequencies between cases (POAG/PACG) for rs1063192 was not found to be statistically significant as given in Table 2. No significant difference in genotype distribution was obtained under genetic model analysis for any of the groups.

**Table 2:** Allele/genotype frequency distributions and genetic model analysis for *CDKN2B* rs3217992and rs1063192among POAG, PACG and controls

Genotype/Allele	POAG n = 313 (%)	Control n = 444 (%)	Chisq value	*p* value	OR(95% CI)
rs3217992					
T	249 (39.77)	399 (44.93)	3.987	0.045*	0.80 (0.65–0.99)
C	377 (60.23)	489 (55.07)	**Ref**	**Ref**	
TT	55 (17.57)	93 (20.95)	4.173	0.074	0.68 (0.45–1.03)
CT	139 (44.41)	213 (47.97)		0.093	0.75 (0.54–1.04)
CC	119 (38.02)	138 (31.08)		**Ref**	
Dominant model	194/119	306/138	3.941	0.047*	0.73 (0.54–0.99)
Recessive model	55/258	93/351	1.329	0.249	0.80 (0.55–1.16)
Codominant model	139/174	213/231	0.800	0.333	0.86 (0.64–1.15)
CA trend under additive model	249/377	399/489	3.796	0.046*	0.80 (0.65–0.99)

rs1063192	POAG n = 313 (%)	Control n = 449 (%)	Chisq value	*p* value	OR(95% CI)
G	178 (28.43)	248 (27.62)	0.122	0.726	1.04 (0.82–1.30)
A	448 (71.57)	650 (72.38)	**Ref**	**Ref**	
GG	21 (6.71)	34 (7.57)	0.917	0.807	0.93 (0.52–1.66)
GA	136 (43.45)	180 (40.09)		0.399	1.13 (0.84–1.53)
AA	156 (49.84)	235 (52.34)		**Ref**	
Dominant model	157/156	214/235	0.460	0.497	1.10 (0.82–1.47)
Recessive model	21/992	34/415	0.205	0.650	0.87 (0.49–1.54)
Co-dominant model	136/177	180/269	0.726	0.354	1.14 (0.85–1.53)
CA trend assuming additive model	178/448	248/650	0.126	0.726	1.04 (0.82–1.30)

rs3217992	PACG n = 147(%)	Control n = 444(%)	Chisq value	*p* value	OR (95% CI)
T	110 (37.41)	399 (44.93)	**5.091**	0.024*	0.73 (0.55–0.96)
C	184 (62.59)	489 (55.07)	**Ref**	**Ref**	
TT	21 (14.29)	93 (20.95)	**4.943**	0.030*	0.53 (0.30–0.94)
CT	68 (46.26)	213 (47.97)		0.189	0.75 (0.50–1.14)
CC	58 (39.45)	138 (31.08)		**Ref**	
Dominant model	89/58	306/138	3.494	0.061	0.69 (0.46–1.01)
Recessive model	21/126	93/315	3.147	0.076	0.62 (0.37–1.05)
Codominant model	68/79	213/231	0.070	0.718	0.93 (0.64–1.35)
CA trend assuming additive model	110/184	399/489	**4.941**	0.030*	0.53 (0.30–0.94)

rs1063192	PACG n = 148(%)	Control n = 449(%)	Chisq value	*p* value	OR (95% CI)
G	70 (23.65)	248 (27.62)	1.794	0.180	0.81 (0.59–1.10)
A	226 (76.35)	650 (72.38)	**Ref**	**Ref**	
GG	9 (6.08)	34 (7.57)	1.904	0.396	0.71 (0.32–1.55)
GA	52 (35.14)	180 (40.09)		0.218	0.78 (0.52–1.15)
AA	87 (58.78)	235 (52.34)		**Ref**	
Dominant model	61/87	214/235	1.861	0.172	0.77 (0.52–1.12)
Recessive model	9/139	34/415	0.370	0.542	0.79 (0.36–1.68)
Codominant model	52/96	180/269	0.951	0.228	0.80 (0.54–1.19)
CA trend assuming additive model	70/226	248/650	1.784	0.181	0.81 (0.59–1.10)

*CA* Cochran Armitage trend test

*p value < 0.05 was considered to be statistically significant and are in bold; Bonferroni corrected *p* value: 0.003 Ref indicates allele/genotype considered as reference while calculating odds ratio and *p* values (in bold)

### Mutant genotype ‘TT’ of rs3217992 confers marginal protection against PACG among male subjects

Stratification of POAG and PACG cases on the basis of gender revealed no significant difference in frequencies of CC and CT genotypes among male as well as female subjects (Table 3). However, TT genotype was overrepresented among control males with respect to PACG cases (*p* = 0.058, OR = 0.36; CI = 0.12–1.03). When the comparison in context to mutant genotype (TT) was done among POAG/PACG females with control female subjects, the study failed to obtain any significant difference in the genotype frequency. Genetic model analysis did not reveal a statistically significant difference in genotypic distribution among primary glaucoma (POAG/PACG) males and females as compared to control subjects (Table 3).

**Table 3:** Gender wise distribution of Genotype frequency and genetic model analysis for *CDKN2B* rs3217992 among POAG and PACG cases with respect to control subjects

Males with POAG and PACG versus control male subjects								
Genotype	POAG n = 200 (%)	Control n = 227 (%)	*p* value	OR(CI)	PACG n = 51 (%)	Control n = 227 (%)	*p* value	OR(CI)
CC	74(37)	66(29.07)	**Ref**		19(37.25)	66(29.07)	**Ref**	
CT	92(46)	113(49.77)	0.145	0.72(0.47–1.11)	27(52.94)	113(49.77)	0.58	0.83(0.42–1.60)
TT	34(17)	48(21.14)	0.101	0.63(0.36–1.09)	5(9.80)	48(21.14)	0.058^a^	0.36(0.12–1.03)
*Genetic models*								
Dominant model	126/74	161/66	0.082	0.69(0.46–1.04)	32/19	161/66	0.253	0.69(0.36–1.30)
Recessive model	92/108	113/114	0.435	0.85(0.58–1.25)	27/24	113/114	0.683	1.13(0.61–2.08)
Additive model	160/240	209/245	0.075	0.78 (0.59–1.02)	37/65	212/244	0.061	0.65 (0.42–1.02)

Females with POAG and PACG versus control female subjects								
Genotype	POAG n = 113 (%)	Control n = 217 (%)	*p* value	OR(CI)	PACG n = 94 (%)	Control n = 217 (%)	*p* value	OR(CI)
CC	45(39.82)	72(33.17)	**Ref**		39(41.48)	72(33.17)	**Ref**	
CT	47(41.59)	101(46.54)	0.255	0.74(0.44–1.23)	43(45.74)	101(46.54)	0.371	0.78(0.46–1.33)
TT	21(18.58)	44(20.27)	0.408	0.76(0.46–1.20)	12(12.76)	44(20.27)	0.072	0.50(0.23–1.06)
*Genetic models*								
Dominant model	68/45	145/72	0.231	0.75(0.46–1.20)	55/39	145/72	0.161	0.70(0.42–1.15)
Recessive model	47/66	101/116	0.391	0.81(0.51–1.21)	43/51	101/116	0.896	0.96(0.59–1.57)
Additive model	89/137	189/245	0.303	0.84(0.60–1.16)	67/121	189/245	0.066	0.71(0.50–1.02)

*OR* odds ratio, *CI* confidence interval

^a^Indicates significant *p* value

### Frequency distribution of rs2157719 T > C and rs4977756 A > G intronic variants of *CDKN2B-AS1* among POAG and PACG cases as compared to controls

Table 4 shows the comparison of allele and genotype frequencies of *CDKN2B-AS1* rs2157719 and rs4977756 intronic polymorphisms. Categorization of two groups was done in a similar fashion as in case of rs3217992 and rs1063192 variants. The genotype count for rs4977756 in controls followed HWE frequency but showed marked deviation for rs2157719. We presume that this deviation might be due to chance factors as other variants were in HWE in controls [31]. The minor allele (C) frequency of rs2157719 was found be significantly higher among controls as compared to cases (POAG/PACG). For rs4977756 minor allele G was almost equally prevalent among POAG cases and controls but it was found to be higher among controls than PACG cases. For rs2157719, significant difference was observed for both allele as well as genotype frequency distribution on comparing POAG and PACG cases with controls as given in Table 4. Since the distribution of genotypes in the population deviates from HWE, the frequency of genotypes was compared by the Cochran–Armitage test for trend assuming additive model [32]. However since our combined sample is in HWE, the allelic and trend statistic were found to be almost equivalent for the combined dataset [32]. After segregating the samples, the trend test revealed association in POAG and PACG (*p* values = 0.028 and 0.004 respectively) but the results were non-significant after Bonferroni correction.

**Table 4:** Allele/Genotype frequency distributions and genetic model analysis for *CDKN2B-AS1* rs2157719 and  rs4977756 among POAG, PACG and controls

Genotype/Allele	POAG n = 312 (%)	Control n = 442 (%)	Chisq value	*p* value	OR(95% CI)
rs2157719					
C	173 (27.72)	292 (33.03)	**4.831**	0.0279*	0.77(0.62–0.97)
T	451 (72.28)	592 (66.96)	**Ref**	**Ref**	
CC	19 (6.09)	65 (14.70)	**14.37**	0.0010*	0.39 (0.22–0.69)
CT	135 (43.27)	162 (36.65)		0.4224	1.13(0.83–1.54)
TT	158 (50.64)	215 (48.64)		**Ref**	
Dominant model	154/158	227/215	0.292	0.5888	0.92(0.69–1.23)
Recessive model	19/293	65/377	**13.72**	0.0002*	0.37(0.22–0.64)
Codominant model	135/177	162/280	3.083	0.0673	1.31(0.98–1.77)
CA trend assuming additive model	173/451	292/592	**4.488**	0.0281*	0.77(0.62–0.97)

rs4977756	POAG n = 313 (%)	Control n = 444 (%)	Chisq value	*p* value	OR(95% CI)
G	165 (26.36)	232 (26.13)	0.010	0.919	1.01(0.80–1.27)
A	461 (73.64)	656 (73.87)	**Ref**	**Ref**	
GG	18 (5.75)	33 (7.43)	1.616		0.80(0.43–1.47)
GA	129 (41.21)	166 (37.39)			1.14(0.84–1.55)
AA	166 (53.04)	245 (55.18)		**Ref**	
Dominant model	147/166	199/245	0.340	0.559	1.09(0.81–1.45)
Recessive model	18/295	33/411	0.826	0.363	0.75(0.41–1.37)
Codominant model	129/184	166/278	0.975	0.287	1.17(0.87–1.57)
Additive model	165/461	232/656	0.010	0.919	1.01(0.80–1.27)

rs2157719	PACG n = 147(%)	Control n = 442(%)	Chisq value	*p* value	OR (95% CI)
C	71 (24.15)	292 (33.03)	**8.164**	0.004*	0.64 (0.47–0.87)
T	223 (75.85)	592 (66.96)	**Ref**	**Ref**	
CC	9 (6.13)	65 (14.70)	**8.305**	0.005*	0.35 (0.16–0.73)
CT	53 (36.05)	162 (36.65)		0.352	0.82 (0.55–1.23)
TT	85 (57.82)	215 (48.64)		**Ref**	
Dominant model	62/85	227/215	**3.72**	0.053*	0.69 (0.47–1.00)
Recessive model	9/138	65/377	**7.398**	0.006*	0.37 (1.83–0.78)
Codominant model	53/94	162/280	0.010	0.896	0.97 (0.66–1.43)
CA trend assuming additive model	71/223	292/592	**7.137**	0.004*	0.64 (0.47–0.87)

rs4977756	PACG n = 147(%)	Control n = 444(%)	Chisq value	*p* value	OR (95% CI)
G	65 (22.11)	232 (26.13)	1.895	0.168	0.80 (0.58–1.09)
A	229 (77.89)	656 (73.87)	**Ref**	**Ref**	
GG	7 (4.76)	33 (7.43)	1.944	0.215	0.58 (0.24–1.36)
GA	51 (34.69)	166 (37.38)		0.407	0.84 (0.56–1.25)
AA	89 (60.54)	245 (55.18)		**Ref**	
Dominant model	58/89	199/245	1.293	0.255	0.80 (0.54–1.17)
Recessive model	7/140	33/411	1.248	0.263	0.62 (0.26–1.43)
Codominant model	51/96	166/278	0.239	0.557	0.88 (0.60–1.31)
CA trend assuming additive model	65/229	232/656	1.85	0.169	0.80 (0.58–1.09)

*p value < 0.05 was considered to be statistically significant and are in bold. Ref indicates allele/genotype considered to be the reference while calculating Odds ratio and is in bold

Genetic data analysis of rs4977756 revealed G allele as the risk allele among POAG cases while A allele was found to confer risk towards PACG, though the distribution of allele frequencies between cases (POAG/PACG) was not found to be statistically significant as given in Table 4.

### CC genotype of rs2157719 confers protection against primary glaucoma (POAG/PACG) among male and female subjects

Subsequent segregation of POAG and PACG cases based on gender (Table 5) revealed a different frequency distribution of genotypes among males and females of two groups i.e. POAG and PACG. A lesser number of CC mutant homozygotes were observed among patients (POAG/PACG) of both genders (Table 5). CC genotype conferred 0.33- and 0.20-fold protection among POAG (*p* = 0.0041) and PACG (*p* = 0.035) males with respect to control males. In the genetic model analysis, recessive model provided 0.29- and 0.22-fold protection against glaucoma (POAG/PACG) progression among male subjects. When the comparison of POAG females was done with healthy female subjects, the study failed to obtain any significant difference in the genotypic frequencies. Genetic model investigation also did not reveal any statistically significant difference in the genotypic distribution among POAG females and control female subjects. In contrast to POAG females, the frequency of CC genotype was significantly different among females having PACG (*p* = 0.029) with respect to control females. The recessive model unveiled 0.34-fold protection against PACG development in females (*p* = 0.033, OR = 0.34; 0.12–0.91) but the results were not significant after Bonferroni correction as shown in Table 5.

**Table 5:** Gender wise distribution of Genotype frequency and genetic model analysis for *CDKN2B-AS1* rs2157719 among POAG and PACG cases with respect to control subjects

Males with POAG and PACG versus control male subjects								
Genotypes	POAG n = 201 (%)	Control n = 226 (%)	*p* value	OR(CI)	PACG n = 52 (%)	Control n = 226 (%)	*p* value	OR(CI)
CC	10(4.9)	34(15.04)	0.0041*	0.33(0.15–0.70)	2(3.84)	34(15.04)	0.035*	0.20(0.04–0.89)
CT	89(44.27)	77(34.07)	0.2	1.30(0.86–1.95)	17(32.69)	77(34.07)	0.43	0.76(0.40–1.47)
TT	102(50.74)	115(50.88)	**Ref**		33(63.46)	115(50.88)	**Ref**	
*Genetic models*								
Dominant model	99/102	111/115	0.97	1.00(0.68–1.47)	19/33	111/115	0.1	0.59(0.32–1.11)
Recessive model	10/191	34/192	0.001*	0.29(0.14–0.61)	2/50	34/192	0.045*	0.22(0.05–0.97)
Additive model	109/293	146/308	0.107	0.78(0.58–1.05)	21/83	146/308	0.017*	0.53(0.31–0.89)

Females with POAG and PACG versus control female subjects								
Genotypes	POAG n = 111 (%)	Control n = 216 (%)	*p* value	OR(CI)	PACG n = 95 (%)	Control n = 216 (%)	*p* value	OR(CI)
CC	9(8.10)	30(13.88)	0.17	0.56(0.25–1.27)	5(5.26)	30(13.88)	0.029*	0.32(0.11–0.89)
CT	48(43.24)	84(38.80)	0.75	1.07(0.66–1.75)	38(40)	84(38.88)	0.64	0.88(0.53–1.47)
TT	54(48.64)	102(47.22)	**Ref**		52(54.73)	102(47.22)	**Ref**	
*Genetic models*								
Dominant model	57/54	114/102	0.80	0.94(0.59–1.49)	43/52	114/102	0.22	0.73(0.45–1.20)
Recessive model	9/102	30/186	0.13	0.54(0.25–1.19)	5/90	30/186	0.033*	0.34(0.12–0.91)
Additive model	66/156	144/288	0.350	0.84(0.59–1.20)	48/142	144/288	0.045*	0.67(0.46–0.99)

*OR* odds ratio, *CI* confidence interval

*Indicates significant *p* value  (in bold). Ref indicates allele/genotype considered to be the reference while calculating Odds ratio and is in bold

### Haplotype and linkage disequilibrium (LD) analysis

**Fig. 3:**
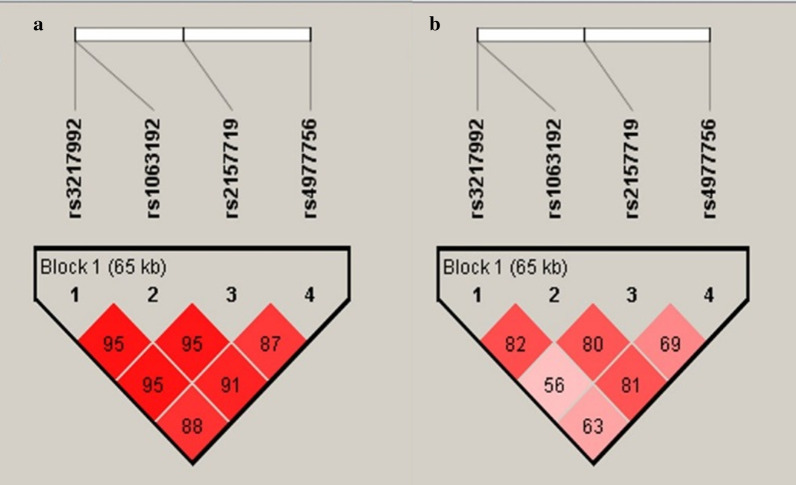
LD plots showing the position of four SNPs of CDKN2B and CDKN2B-AS1 genes and D′ values observed in cases (**a**) and controls (**b**)

The distribution of haplotype frequency and measure of linkage disequilibrium (LD) for primary glaucoma cases and controls is shown in Table 6. LD analysis revealed that SNPs of *CDKN2B* (rs3217992, rs1063192) and *CDKN2B-AS1* (rs2157719, rs4977756) were in very high LD among cases (D′ = 0.95, r^2^ = 0.87) and controls (D′ = 0.87, r^2^ = 0.75) respectively. Moreover, rs1063192 was also found to be in strong LD with rs2157719 (D′ = 0.95, r^2^ = 0.87) and rs4977756 (D′ = 0.91, r^2^ = 0.81). However, controls were observed to be in slightly lower LD as compared to cases for both variants of the two genes for rs3217992/rs1063192 (D′ = 0.82, r^2^ = 0.21) and rs2157719/rs4977756 (D′ = 0.69, r^2^ = 0.34) (Fig. 3). Haplotype analysis unveiled that CATA haplotype conferred 1.61-fold risk for primary glaucoma (*p* ≤ 0.0001; OR = 1.61(CI = 1.31–1.98) as given in Table 6.

**Table 6:** Distribution of haplotypes observed for polymorphisms of the *CDKN2B *and *CDKN2B-AS1* genes in total primary glaucoma cases and controls

Haplotype^a^	Haplotype frequency	Cases	Controls	*p* value	OR(95% CI)
Frequency in total glaucoma cases versus controls					
TATA	0.37	0.37	0.36	0.677	1.04(0.86–1.25)
CATA	0.28	0.33	0.23	< 0.0001*	1.61(1.31–1.98)
CGCG	0.20	0.20	0.19	0.137	1.18(0.94–1.48)

^a^Order of SNPs in *CDKN2B* and *CDKN2B-AS* haplotypes: rs3217992 C/T, rs1063192 A/G, rs2157719 T/C, rs4977756 A/G

*indicates significant* p* value

### Meta-analysis

#### Study Characteristics (rs1063192 A > G)

Total 125 articles were retrieved by literature survey. After examining all publications, 18 related articles were included along with the current study. Therefore, the current meta-analysis was performed with 19 total studies. This meta-analysis comprised all articles representing total cases (n = 13,253) and controls (n = 34,615) from 2011 to 2019. All included studies dealt with association between 4 sequence variants of *CDKN2B/AS1* genes and POAG. Table 7 indicates the main features of all the studies. For meta- analysis of rs1063192, 18 studies were included. Out of 18, 6 studies represent POAG patients belonging to Caucasian ancestry. Among these 6, 4 studies were conducted in Australia, 1 in Saudi Arabia, while 1 in USA. Remaining 8 studies targeted Asian population, in which 3 studies were from Japan, 1 from China, 1 from Pakistan and 3, including the current study were reported from Indian population. 3 other studies represented African ancestry, in which 2 were conducted in USA and 1 in South Africa. 1 study which was conducted in West Indies 2012, assessed mixed population (Afro-Carribean). Genotypic methods for studies included PCR–RFLP, TaqMan and Seq-illumina. The genotypic distribution among controls was found to be consistent with HWE in all studies.

**Table 7:** Characteristics of the included POAG studies in meta-analysis

SNP	Year	Author	Country	Ethnicity	Genotyping	Case	Con	OR	*p* value	Source	Association (yes/no)
rs1063192 (A > G)											
	2016	Ng	Australia	Caucasian	Illumina	2241	3176	1.43 (1.32–1.55)	3.76 × 10^−18^	PB	Yes
	2016	Abu-Amero	Saudi Arabia	Caucasian	Taqman	87	94	0.94 (0.54–1.59)	0.799	HB	No
	2015	Burdon	Australia	Caucasian	PCR	67	1919	1.47 (1.03–2.11)	0.033	PB	Yes
	2015	Chen	China	Asian (Han Chinese)	Sequenom	1157	934	0.85 (0.72–1.00)	0.047	HB	Yes
	2015	Williams	South African	African	Illumina	215	214	2.58 (0.21–31.4)	0.4585	HB	No
	2015	Philomena-din	India	Asian	PCR	97	371	Nd	0.95	HB	No
	2012	Mabuchi	Japan	Asian	RT PCR	425	191	Nd	Nd	HB	No
	2014	Michael	Pakistan	Asian	Taqman	268	233	0.91 (0.68–1.21)	0.48	HB	No
	2013	Liu	USA	African American	RT PCR	1150	999	0.85 (0.67–1.06)	0.15	PB	No
	2013	Liu	USA	West African	RT PCR	483	593	0.98	N/A	PB	No
	2012	Cao	West Indies	Afro- Carribean	PCR	272	165	0.39 (0.22–0.69)	0.0008	PB	Yes
	2012	Osman (GWAS)	Japan	Asian	PCR	1393	6591	0.79 (0.71–0.86)	5.68 × 10^−5^	PB	Yes
	2012	Osman	Japan	Asian	PCR	1800	7207	0.73 (0.64–0.82)	1.73 × 10^−7^	PB	Yes
	2012	Dimasi	Australia	Caucasian	Sequemass	876	883	0.74 (0.64–0.85)	2.2 × 10^−5^	PB	Yes
	2012	Takamoto	Japan	Asian	Taqman	183	514	1.41 (1.02–1.96)	0.0381	HB	Yes
	2011	Fan	USA	US Caucasian	Taqman	389	308	0.73 (0.58–0.90)	0.0045	PB	Yes
	2011	Burdon	Australia	Caucasian	Illumina	892	4582	1.44 (1.28- 1.61)	7.46 × 10^−10^	PB	Yes
	2014	Vishal	India	Asian (East Indians)	Illumina	700	708	0.98 (0.77–1.26)	0.9	HB	No
	2019	Present study	India	Asian	RT-PCR	313	449	1.04 (0.82–1.30)	0.726	HB	No
rs2157719 (A > G)											
	2016	Abu-Amero	Saudi Arabia	Caucasian	Taqman	85	95	1.2 (0.73–2.07)	0.429	HB	No
	2015	Chen	China	Asian	Sequenom	1157	934	0.64 (0.52–0.79)	3.528 × 10^−5^	HB	Yes
	2017	Shiga	Japan	Asian	Japonica Array	565	1104	1.61 (1.29–2.00)	1.42E10^−5^	PB	Yes
	2014	Vishal	India	Asian (East Indian)	Illumina	700	708	0.96 (0.75–1.23)	0.76	HB	No
	2019	Present Study	India	Asian	RT-PCR	312	442	0.77 (0.62–0.97)	0.0279	HB	Yes
rs4977756 (A > G)											
	2014	Michael	Pakistan	Asian	Taqman	268	233	0.92 (0.68–1.23)	0.55	HB	No
	2013	Liu	USA	African American	Real time PCR	1150	999	0.91 (0.81–1.04)	0.16	PB	No
	2013	Liu	USA	West African	Real time PCR	483	593	1.07 (0.90–1.29)	0.44	PB	No
	2017	Yoshikawa	Japan	Asian	Taqman	736	2723	0.77	3.2 X 10^−4^	HB	Yes
	2011	Burdon	Australia	Caucasian	Illumina	892	4582	1.33 (1.19–1.48)	4.19 × 10^−7^	PB	Yes
	2016	Ng	Australia	Caucasian	Illumina	2241	3176	1.40 (1.26–1.52)	1.97 × 10^−16^	HB	Yes
	2015	Chen	China	Asian (Han Chinese)	Sequenom	1157	934	0.89 (0.76–1.04)	0.128	HB	No
	2012	Cao	West Indies	Afro-Carribean	PCR	272	165	0.89 (0.67–1.20)	0.4507	PB	No
	2014	Vishal	India	Asian	Illumina	700	708	0.99 (0.78–1.27)	0.95	HB	No
	2015	Burdon	Australia	Caucasian	PCR	67	1919	1.52 (1.05–2.20)	0.027	PB	Yes
	2015	Williams	South African	African	Illumina	215	214	1.14 (0.59–2.18)	0.6974	HB	No
	2019	Present study	India	Asian	RT-PCR	313	444	0.80 (0.58–1.09)	0.168	HB	No
rs3217992 (C > T)											
	2011	Burdon	Australia	Caucasian	Illumina	892	4582	1.22 (1.09- 1.37)	5.04 × 10^−4^	PB	Yes
	2012	Takamoto	Japan	Asian	Digitag2 Assay	183	514	1.53 (1.20–1.96)	6.35 × 10^–4^	HB	Yes
	2014	Vishal	India	Asian (East Indians)	Illumina	700	708	1.15 (0.93–1.42)	0.21	HB	No
	2019	Present study	India	Asian	RT-PCR	313	444	0.80 (0.65–0.99	0.045	HB	Yes

*PB* population based, *HB* hospital based, *CON* control, *OR* odds ratio

#### Study characteristics (rs3217992 C > T)

Meta-analysis of rs3217992 comprised of 4 studies including total 2088 affected and 10,266 unaffected subjects. It included 2 studies from Indian population (including the current study). 1 report represented Caucasians and 1 from Japan. All the essential information of included studies is given in Table 7. Diverse genotyping techniques used in selected studies were TaqMan, Seq-illumina and PCR–RFLP method. The genotypes followed HWE in controls in all studies.

#### Meta-analysis of rs1063192 and rs3217992

**Fig. 4:**
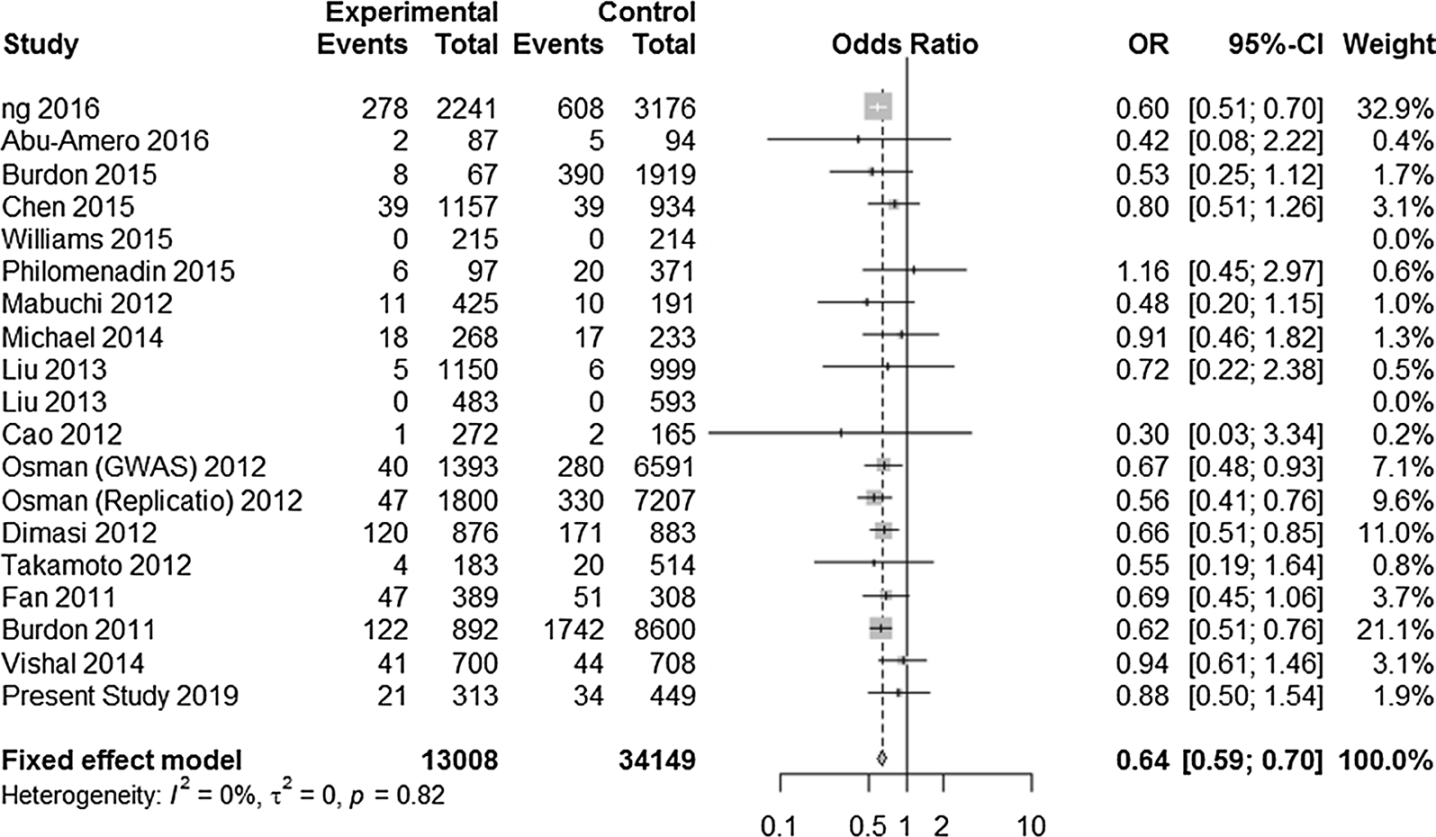
Forest plot depicting association between *CDKN2B* variant (rs1063192) under recessive model (GG vs AA + GA) and POAG. The black horizontal lines correspond to the 95% CI and the position of black small squares to the OR per study. The size of the square is proportional to the relative weight of that study in % to compute the overall OR (black diamond). The width of the diamond represents the 95% CI of the overall OR. This study has revealed statistically significant association between this SNP and POAG. This meta-analysis illustrates protection conferred by GG genotype against glaucoma progression with overall OR = 0.64 (*p* ≤ 0.0001) with no heterogeneity (*I*^2^ = 0%; *p* = 0.82) under fixed effect model

Main features of results of meta-analysis for rs1063192 are shown in Table 8. Under random effect model, overall a statistically significant association was found between this sequence variant and POAG risk in current meta-analysis. Dominant model (GG + GA vs AA) revealed significant association with OR = 0.76; *p* < 0.0001, with substantial heterogeneity, I^2^ = 64.6%. In recessive model (GG vs GA + AA), highly statistically significant association was observed with overall protective effect of GG genotype from disease progression, OR = 0.64; *p* < 0.00001, with no heterogeneity, I^2^ = 0% as shown in Fig. 4. When we assessed this SNP under heterozygous model (GA vs GG + AA), moderate heterogeneity (I^2^ = 43.6%) with OR = 0.84 (95% CI = 0.80–0.88) was observed (Table 8). The sensitivity analysis for recessive model was done, which investigated the influence of a single study on the overall risk estimate by omitting one study at each turn. This yielded a range of OR from 0.6362 (95% CI 0.5818–0.6956); *p* < 0.0001 to 0.6625 (95% CI 0.5955–0.7369); *p* < 0.0001. This suggested that exclusion of any single study did not altered the overall combined OR.

**Table 8:** Meta-analysis of association between *CDKN2B* rs1063192 polymorphism and the risk of POAG

Genetic models rs1063192 A > G	Model (Fixed effect/random effect) OR (overall)	95% CI	*p* value (overall)	Heterogenity (*p* value)	*I*^2^ (%)	Ʈ	Begg test *p* value	Egger test *p* value
Dominant model	Random effect 0.76	0.69–0.84	< 0.0001	< 0.01	65	0.0214	0.1598	0.3837
Recessive model	Fixed effect 0.64	0.59–0.70	< 0.0001	0.82	0	0	0.8048	0.2997
Heterozygous model	Fixed effect 0.84	0.80–0.88	< 0.0001	0.03	43.6	0.0091	0.383	0.348

#### Publication bias for rs3217992

**Fig. 5:**
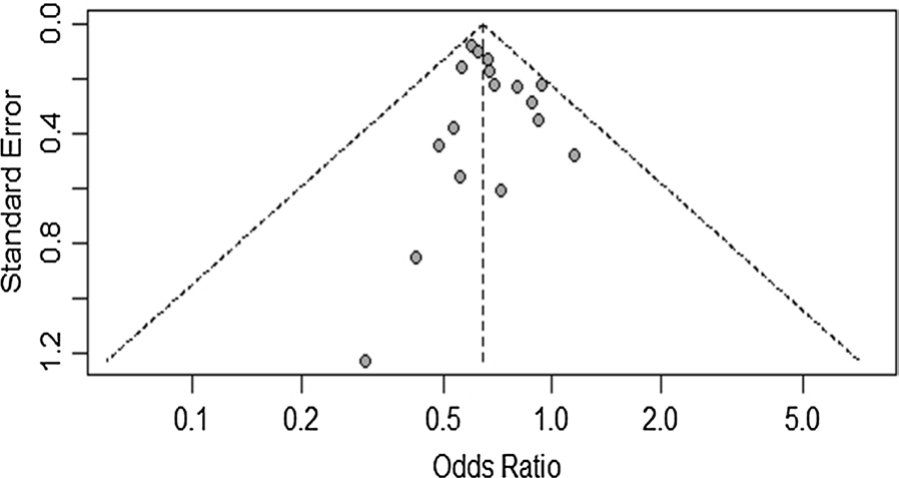
Funnel plot of included case–control studies under fixed effect model showing  no publication bias and hence symmetry in funnel plot

Visual inspection of the funnel plot did not identify obvious asymmetry. The Begg (*p* = 0.383) and Egger’s test (*p* = 0.348) for funnel plot did not reveal any asymmetry in funnel plot (Fig. 5).

**Fig. 6:**
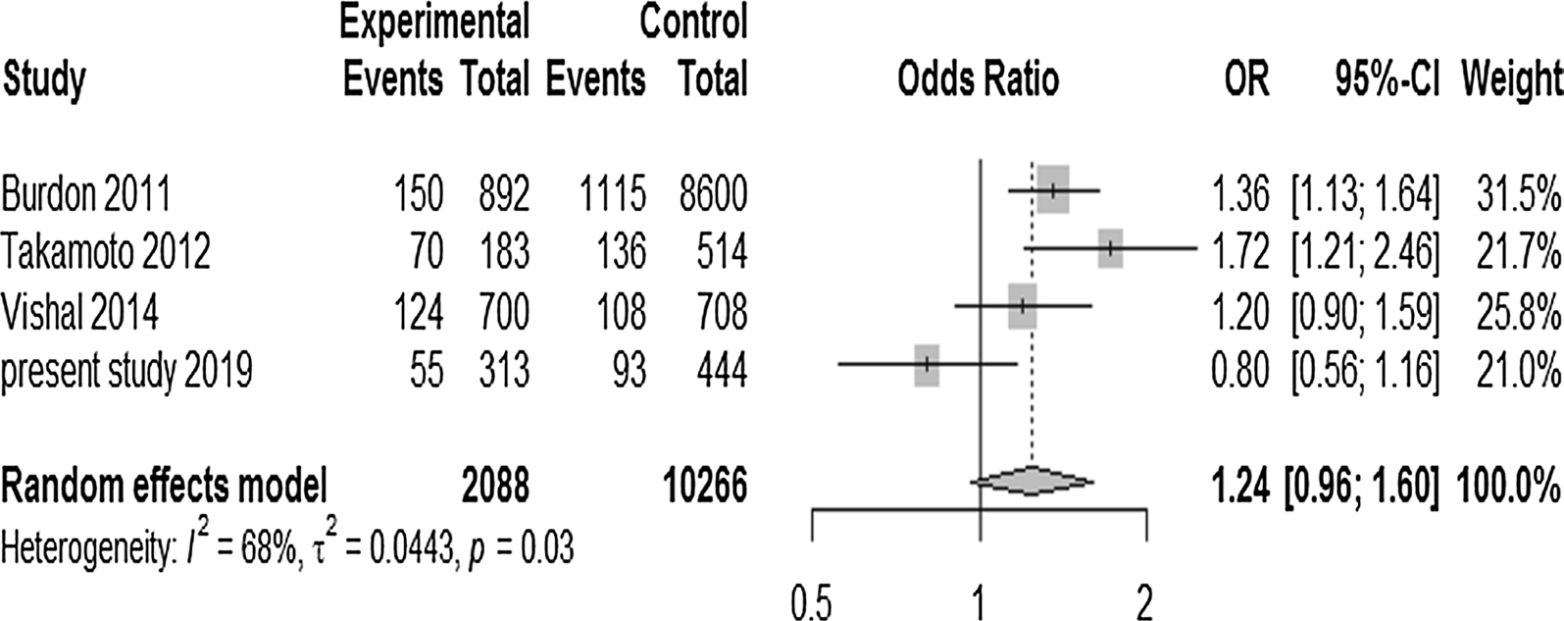
Forest plot showing association between *CDKN2B* variant (rs3217992) under recessive model (TT vs CC + CT) and POAG. Symbols and conventions indicated here are same as mentioned earlier in Fig. 4. This meta-analysis did not unveil any association between this SNP and POAG with overall OR = 1.24 (*p* = 0.097) with substantial heterogeneity (*I*^2^ = 67.8%; *p* = 0.03) under random effect model

For rs3217992, the pooled OR ranged between 1.03 and 1.23, under various genetic model (Dominant, Recessive (Fig. 6), Heterozygous) analysis as given in Table 9 with 95% CI ranging from 0.88 to 1.59 with overall non-significant *p* value. The sensitivity analysis for recessive model was done, which investigated the overall risk estimate by omitting the present study. This yielded a range of OR: 1.3698 (95% CI 1.1611–1.6160); *p* < 0.0001 to 0.6625 (95% CI 0.5955–0.7369); *p* = 0.0002 with 19.2% heterogeneity which differed from pooled OR: 1.2393 (95% CI 0.9612–1.5977), *p* = 0.0979 with 67.8% heterogeneity (Fig. 6). This suggested that exclusion of present single study obviously altered the overall combined significant association and gave maximum heterogeneity.

**Table 9:** Meta-analysis of association between *CDKN2B* rs3217992 polymorphism and the risk of POAG

Genetic models rs3217992 A > G	Model (Fixed effect/random effect) OR (overall)	95% CI	*p* value (overall)	Heterogenity (*p* value)	*I*^2^ (%)	Ʈ	Begg test *p* value	Egger test *p* value
Dominant model	Random effect 1.16	0.88–1.52	0.2779	< 0.01	78.5	0.056	NA	NA
Recessive model	Random effect 1.23	0.96–1.59	0.0979	0.03	67.8	0.044	NA	NA
Heterozygous model	Fixed effect 1.03	0.94–1.17	0.3178	0.41	0	0	NA	NA

#### Publication bias for rs1063192

Visual inspection of the funnel plot did not identify obvious asymmetry. The Egger test for funnel plot asymmetry was not performed as the power of this test is too low to distinguish chance from real asymmetry when the meta-analysis included less than 10 studies.

#### Study characteristics (rs2157719A > G)

Samples from 5 studies (cases = 2819 and controls = 3283) were pooled together to calculate the association of rs2157719 with POAG. It included 1 Caucasian and 4 Asian studies. Out of 4 Asian studies, 2 studies were from Indian population including the current study, 1 report was from China and 1 from Japan as given in Table 7. TaqMan, Sequenom, Japonica, and Illumina assays were used to genotype samples and genotypic distribution in the studied controls were consistent with HWE.

#### Study characteristics (rs4977756 A > G)

Total 12 studies were short listed for the meta-analysis of rs4977756 to find out overall genotypic association between this variant and POAG. Total 8739 POAG patients and 21,174 unaffected healthy age and gender matched controls were involved. Meta-analysis contained 3 studies on Caucasians, 4 reports on African ancestry, 5 studies from Asians (out of which 1 was conducted in Japan, 1 in China, 1 report from Pakistan and 2 from India, including current study) as represented in Table 7. Genotyping techniques included TaqMan chemistry, Seq-Illumina, Sequenom assay, and PCR–RFLP methods. The genotypic distribution in all studied controls were consistent with HWE.

#### Meta-analysis of rs2157719 and rs4977756

**Fig. 7:**
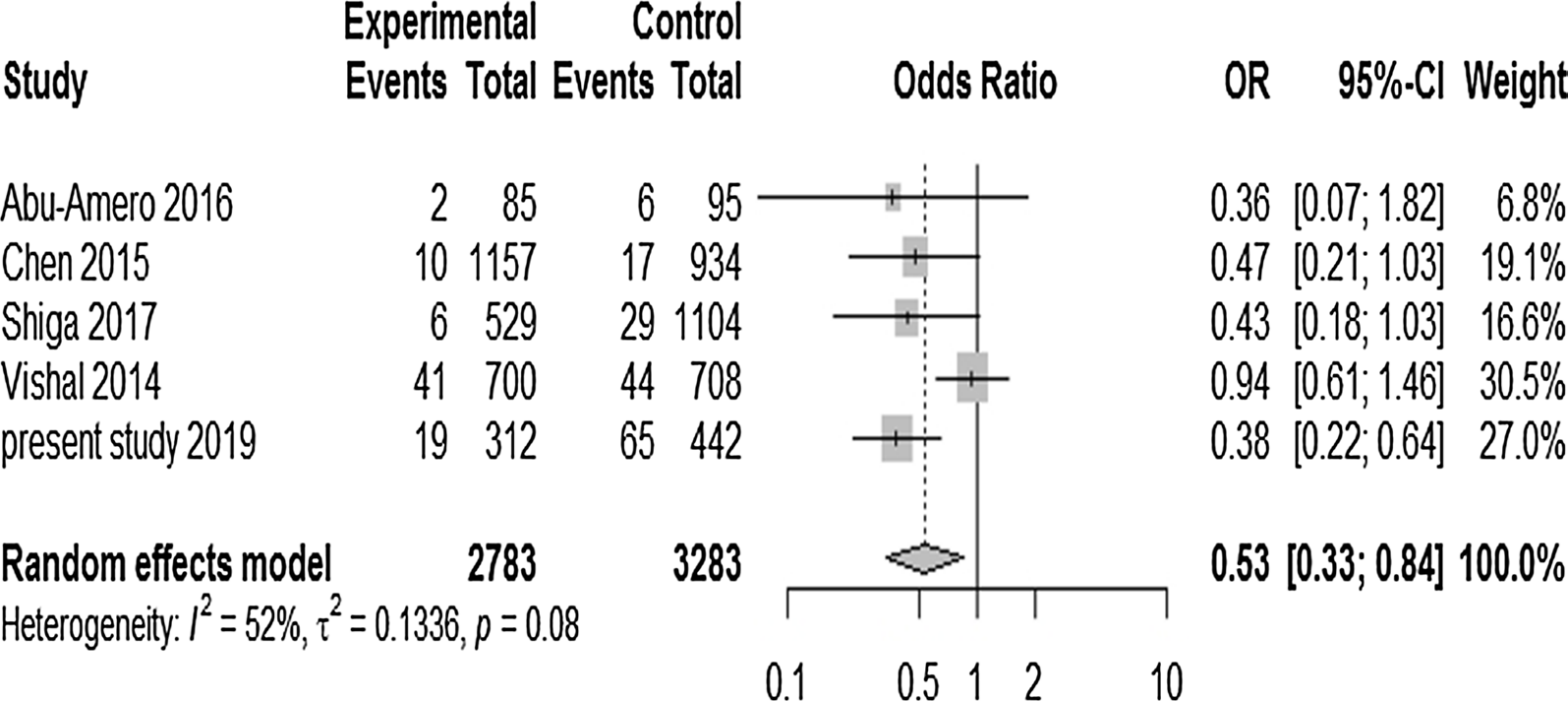
Forest plot indicating association between *CDKN2B-AS1* variant (rs2157719) under recessive model (GG vs GA + AA) and POAG. Symbols and conventions indicated here are same as mentioned earlier in Fig. 4. This meta-analysis unveiled highly significant association between this polymorphism and POAG with overall OR = 0.53 (*p* = 0.007) with substantial heterogeneity (*I*^*2*^ = 52%; *p* = 0.08) under random effect model

Main features of results of meta-analysis for rs2157719 are given in Table 10. Under random effect model, overall a statistically significant association was found between this sequence variant and POAG risk in current meta-analysis under various genetic models (dominant, recessive (Fig. 7) and heterozygous model) (*p* = 0.01, 0.007, < 0.0001 respectively) with pooled OR ranging from 0.46 to 0.77), showing overall protection against POAG as given in Table 10 and Fig. 7. The sensitivity analysis for recessive model was done, which investigated the huge impact of a single study conducted by Vishal et al. in 2014 [26] on the overall risk estimate (OR = 0.4048 (95% CI 0.2758–0.5943); *p* < 0.0001) and hence conferring maximum heterogeneity to the data. After omitting this single study there was no heterogeneity (I^2^ = 0%), suggesting that exclusion of this single study altered the overall combined OR.

**Table 10:** Meta-analysis of association between *CDKN2B-AS1* rs2157719 polymorphism and the risk of POAG

Genetic models rs2157719 A > G	Model (Fixed effect/random effect) OR (overall)	95% CI	*p* value (overall)	Heterogenity (*p* value)	*I*^2^ (%)	**Ʈ**	Begg test *p* value	Egger test *p* value
Dominant model	Random effect 0.77	0.64–0.94	0.01	0.04	59.4	0.02	NA	NA
Recessive model	Random effect 0.52	0.33–0.83	0.007	0.08	51.7	0	NA	NA
Heterozygous model	Random effect 0.46	0.34–0.63	< 0.0001	< 0.01	84.5	0.0091	NA	NA

#### Publication bias

Visual inspection of the funnel plot did not identify obvious asymmetry. The Egger test for funnel plot asymmetry was not performed as the power of this test was too low to distinguish chance from real asymmetry when the meta-analysis included less than 10 studies.

**Fig. 8:**
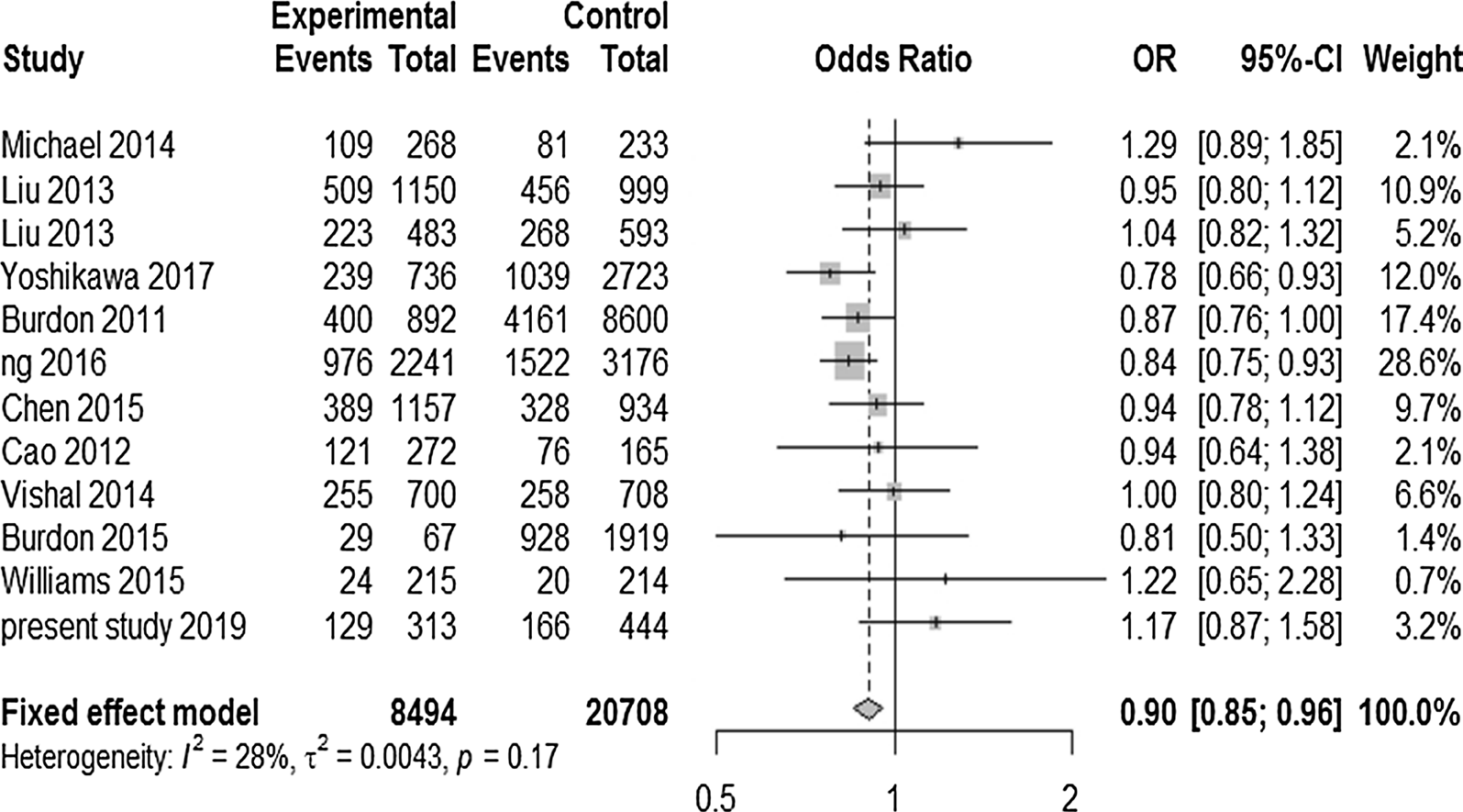
Forest plot depicting association between *CDKN2B-AS1* variant (rs4977756) under heterozygous model (GA vs AA + GG) and POAG. Symbols and conventions indicated here are same as mentioned earlier in Fig. 4. Current meta-analysis revealed statistically significant association between this sequence variant and POAG. This analysis has showed overall OR = 0.90 (*p* = 0.0005) with low heterogeneity (*I*^2^ = 28%; *p* = 0.17) under fixed effect model

For rs4977756, the pooled OR was 0.85, under various genetic model (Dominant, Recessive) analysis as given in Table 11 with 95% CI ranging from 0.75 to 0.97 with overall highly statistically significant *p* value (Table 11). Comparison of heterozygous GA with other two genotypes (GG + AA) among total cases and controls also unveiled protection against POAG with OR = 0.90 (95% CI 0.85–0.95) and highly significant *p* = 0.0005 with very low heterogeneity (I^2^ = 27.9%) among all included studies in meta-analysis as shown in Fig. 8. The sensitivity analysis for the overall risk estimate by omitting one study at a time, yielded OR: 0.9033 (95% CI 0.8532–0.9564) with I^2^ = 27.9%. This suggested that exclusion of single study obviously did not alter the overall combined significant association.

**Table 11:** Meta-analysis of association between *CDKN2B-AS1* rs4977756 polymorphism and the risk of POAG

Genetic models rs4977756A > G	Model (Fixed effect/random effect) OR (overall)	95% CI	*p* value (overall)	Heterogenity (*p* value)	*I*^2^ (%)	Ʈ	Begg test *p* value	Egger test *p* value
Dominant model	Random effect 0.85	0.75–0.97	0.0182	< 0.01	74.1	0.031	0.217	0.095
Recessive model	Fixed effect 0.85	0.75–0.97	0.0182	0.10	74.1	0.031	0.217	0.095
Heterozygous model	Fixed effect 0.90	0.85–0.95	0.0005	0.17	27.9	0.004	0.099	0.026

##### Publication bias

**Fig. 9:**
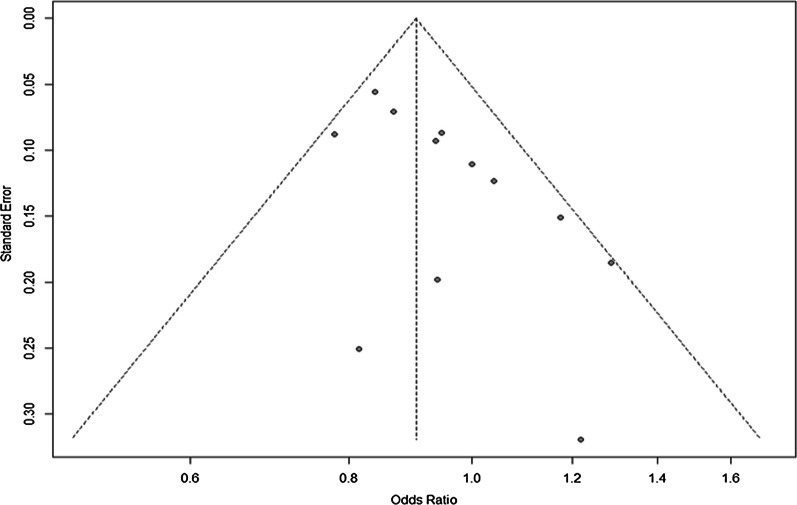
Funnel plot of included case–control studies under fixed effect model assessing no publication bias and hence symmetry

Visual inspection of the funnel plot did not identify obvious asymmetry. The Begg (*p* = 0.217) and Egger’s test (*p* = 0.095) for funnel plot did not reveal any asymmetry in funnel plot as given in Fig. 9.

## Discussion

*INK4* locus at chromosome 9p21 has been implicated in affecting genetic susceptibility to glaucoma in different populations. The present study is first of its kind in North India to investigate the role of variants in *CDKN2B* (rs1063192, rs3217992) and *CDKN2B-AS1* (rs2157719, rs4977756) genes located at the *INK4* locus as genetic risk factors for POAG and PACG. *CDKN2B* is cyclin dependent kinase inhibitor gene that influences cell proliferation and senescence in all cell types [33, 34] including retinal ganglion cells (RGCs). It is reported to be upregulated in response to elevated IOP in animal model of glaucoma suggesting apoptosis of RGCs may be promoted due to altered expression of *CDKN2B* and cause glaucomatous visual field loss [8]. Various studies reveal that single nucleotide polymorphisms in *CDKN2B* can alter its function [16]. In one such study conducted by Pasquale et al. [17] 10 SNPs at *CDKN2B* were screened in a retrospective observational case series and rs3217992 in *CDKN2B* was found to be significantly associated with glaucoma (*p* = 3.34 × 10^−15^). The study included Caucasian POAG patients that presented increased disease risk with larger CDR (*p* = 4.74 × 10^−4^) despite lower IOP in presence of the minor allele (T). Earlier GWA studies have also reported significant association of rs3217992 with POAG in the Japanese and Caucasians [12, 35, 36]. In initial analysis our findings are in line with the previous findings, however in our population T allele confers protection as POAG cases were found to have significantly lower frequency of the minor allele (T) of rs3217992 variant (39.77%) as compared to controls (44.93%) (*p* = 0.045). Same relation was observed in PACG, with T allele being significantly lower among cases as compared to controls (*p* = 0.024). This was in contradiction to the previous finding [17, 35] and points towards different genetic structure among various ethnic groups. Further stratification of the data unveiled the ability of TT genotype to confer 0.36-fold protection towards progression of PACG in males, although we could not achieve same significant association after applying correction for multiple testing. No significant difference was observed in the female population, although the overall risk of developing glaucoma is reported to be higher in older women experiencing menopause compared to males which has been attributed to estrogen specific protection in younger females [37].

Another variant, rs1063192 (A > G) resides in the 3′UTR of *CDKN2B* gene and is strongly correlated with (CDR in a Japanese group [38]. A previous Japanese GWAS reported a strong association of the variant with POAG [(*p* = 5.2 × 10^−11^); (OR = 0.75)] [14]. The association reports of this SNP with POAG has been quite consistent in various Caucasian studies [9, 12, 36, 39, 40]. Analysis in Afro-Caribbean population including a total of 437 unrelated subjects from Barbados family study of open angle glaucoma also found rs1063192 to be significantly associated with POAG (*p* = 0.0008) [16]. Yet in the present study, rs1063192 failed to show any significant association with either disease form. A study from African group also suggested no association of the variant with glaucoma contrary to the reports from the Caucasian groups. In that study, the researchers included two groups, African-Americans and Ghanaians and analyzed 57 SNPs in five loci including *CDKN2B/CDKN2B-AS1, TMCO1, CAV1/CAV2*, chromosome 8q22 intergenic region, and *SIX1/SIX6.* rs10120688 in *CDKN2B-AS1* region was found to be associated with POAG in African Americans (*p* = 0.0020) [41]. According to the HapMap data, this variant is in strong LD with rs2157719 (D′ = 0.99, r^2^ = 0.70), rs1063192 (D′ = 0.97, r^2^ = 0.68) and rs4977756 (D′ = 0.49, r^2^ = 0.67) in Gujarati Indian population. Hence, it is difficult to predict the true functional variant. Nonetheless, in the South Indian population, rs1063192 was associated with one of the endophenotypes of POAG. The researchers studied 97 POAG cases and 371 controls from South India and found C allele of rs1063192 to be associated with decreased axial length in controls suggesting a decreased risk for POAG [27]. Another gene of interest located at *INK4* locus is *CDKN2B-AS1* which encodes for an antisense RNA that regulates expression of *CDKN2A* and *CDKN2B* via forming transcript complexes with polycomb proteins. The antisense region controls the expression of inhibitor genes negatively. Depletion of *ANRIL* was observed to cause increased expression of *CDKN2B* gene confirming that ANRIL binds SUZ12 (one of polycomb protein complexes 2) and regulates *CDKN2B* [40]. The corresponding change in cyclin dependent kinase could affect ganglion cell apoptosis. Due to its suggestive role in ganglion cell apoptosis, the gene has been of special interest in GWASs of various ethnicities including US-Caucasians, Asians, Africans and Europeans [13–15, 19, 35].

rs2157719 (T > C), located in *CDKN2B-AS1* has been reported to be associated with exfoliation glaucoma in Caucasian population [15]. A Japanese group recently did genotype–phenotype analysis and revealed significant correlations between the variant and decreased IOP (β = − 6.89 mmHg, dominant model *p* = 1.70E−04) [42]. Ozel and co-workers also reported a significant association of rs4977756 and rs2157719 at *CDKN2B-AS1* with IOP in a meta-analysis in > 6000 subjects of European ancestry collected in three datasets: NEIGHBOR, GLAUGEN and AMD-MMAP [42, 43]. Case–control replication analyses by Yoshikawa and co-workers also yielded strong association of the rs4977756 with POAG in a Japanese population [(*p* = 3.2 × 10^−4^); OR = 0.77(CI = 0.68–0.86)] [22]. The present study also found significant association of rs2157719 with primary glaucoma on using CA trend test for association. The advantage of the CA trend test is that it is not dependent on the HWE assumption. Moreover, since three SNPs followed HWE, and genotyping errors were ruled out to the best of our ability, we can assume that the controls were representative of the population targeted in the study. Sex-based data stratification further revealed protection towards progression of both disease forms conferred by CC genotype among males. Similar results were obtained in PACG females where CC genotype provided protection against developing the disease but not in POAG females. Even though the observed association did not survive Bonferroni correction, it should not be rejected out rightly. Since the bonferroni correction is overly conservative as it assumes independence among the tests considered, we would be precautious to reject the positive association with rs321792 and rs2157719. More so because the SNPs tested in the current study are in strong LD and this may lead to correlation among the tests [44]. rs4977756 (A > G) failed to show association in our study. The results for this variant have been inconsistent; a Brazilian study denied significant association with glaucoma [45]. Cao et al. also didn’t find any significant association of this SNP with POAG (*p* = 0.4507) in Afro-Caribbean population [16].

Nominal association of rs4977756 with NTG was observed in African Americans (*p* = 0.04) but not in West African population [41]. Ng et al. [40] also reported strong association of the variant with POAG in Australians, especially in the advanced cases. They further established a stronger relation of SNP with POAG in females with OR difference between male and female being statistically significant at *p* = 1.73 × 10^−4^ [40]. However, no such predisposition in the females was seen in our study group for this variant.

Previous findings in India and Pakistan have been consistent in terms that no association was observed between glaucoma and *INK4* locus [26, 46]. A study in the East Indians included all 4 SNPs targeted in this study and found only nominal association of rs1011970 (*p* = 0.048) with POAG and rs10120688 (*p* = 0.048) in NTG patients (IOP < 21 mm of Hg) thereby suggesting lack of significant genetic association of 9p21 locus with POAG [26]. This is in contrast to our findings and further reiterates the heterogeneous nature of populations in the Indian subcontinent due to genetic admixture.

Since there is a significant difference in the results of various studied populations worldwide, we did a meta-analysis to measure the overall association between these variants and risk of primary open angle progression. Pooled analysis on POAG cases and controls revealed a significant association between rs1063192, rs2157719, rs4977756 and POAG except rs3217992. Another meta-analysis, conducted by Hu et al. [47] in 2017 on 11,316 cases and 24,055 controls evaluated *CDKN2B* rs1063192 and it was found to be associated with decreased risk of glaucoma. Our findings were also in the concordance with previously done meta-analysis [47], rs1063192 was observed to be associated with POAG under fixed and random effect model with pooled OR ranging from 0.64 to 0.84 under dominant, heterozygous, recessive genetic model analysis with overall *p* ≤ 0.0001. Meta-analysis of rs2147719 and rs4977756 also showed statistically significant association of these SNPs with decreased risk of POAG progression with pooled OR ranging from 0.46 to 0.77 and 0.85 to 0.90 respectively, under various genetic models. rs3217992 did not give any association in meta-analysis although found to be associated with primary glaucoma in genetic association analysis in the current study. Whether the association obtained with rs3217992 in the present study represents real cause-effect model relationship as its not supported by meta-analysis should be further investigated. Significant association in some studies could indicate these sequence variants to be a risk factor in certain ethnic groups, which requires more data from different populations to do a meta-analysis and correlate it with ethnicity.

## Conclusion

In conclusion, the present study points towards significant association between rs3217992 and rs2157719 with primary glaucoma. The association with rs2157719 with PACG was strong enough to survive Bonferroni correction for multiple testing. In an updated meta-analysis for *INK4* variants significant association was observed with POAG with rs1063192, rs2157719 and rs4977756. Large scale sequencing of the *INK4* locus may reasonably detect true functional and rare variants while in vitro and in vivo studies may further assess the functional relevance of these variants in pathogenesis of primary glaucoma.

## Supplementary Information

**Additional file 1: Table S1**. The Newcastle-Ottawa Scale for the assessment of case-control studies included in the meta-analysis.

## Data Availability

The datasets generated and/or analyzed in the North Indian Punjabi glaucomatous population are included in the manuscript. Any additional information is available upon request from the corresponding author.

## Abbreviations

*CDKN2B*/*CDKN2A*: Cyclic Dependent Kinase Inhibitor2B/Cyclic Dependent Kinase Inhibitor2A
*CDKN2B-AS1*: Cyclic Dependent Kinase Inhibitor2B-Antisense1
RGC: Retinal Ganglionic Cell
GWAS: Genome Wide Association Study
POAG: Primary Open Angle Glaucoma
PACG: Primary Angle Closure Glaucoma
SNP: Single Nucleotide Polymorphism
ANRIL: Antisense Non-coding RNA in the* INK4* locus
VCDR: Vertical Cup Disc ratio
3′UTR: 3′Untranslated Region
IOP: Intra-Ocular Pressure
OCT: Optical Coherence Tomography
SITA: Swedish Interactive Thresholding Algorithm
EDTA: Ethyelene DdiamineTetraacetic Acid
SD: Standard Deviation
OR: Odds Ratio
CI: Confidence Interval
LD: Linkage Disequilibrium
NOS: Newcastle–Ottawa quality assessment Scale
