# Supplementary material for: Genetic association between CDKN2B/CDKN2B-AS1 gene polymorphisms with primary glaucoma in a North Indian cohort: an original study and an updated meta-analysis
Source: BMC Med Genomics. 2021 Jan 4;14:1. doi: 10.1186/s12920-020-00855-1 (PMC7780652; doi:10.1186/s12920-020-00855-1)
Supplement: Supplementary file 1 — Additional file 1: Table S1. The Newcastle-Ottawa Scale for the assessment of case-control studies included in the meta-analysis. [file 12920_2020_855_MOESM1_ESM.docx]

**Table S1**: The Newcastle-Ottawa Scale for the assessment of case-control studies included in the meta-analysis

| **Year** | **Author** | **Selection** | | | | **Comparability** | **Outcome/Exposure** | | | **Total Score** |
| --- | --- | --- | --- | --- | --- | --- | --- | --- | --- | --- |
|  |  | **Case defined adequately** | **Representativeness of the cases** | **Selection of controls** | **Definition of controls** | **Study controls for age/gender and ethnicity** | **Ascertainment of exposure** | **Same selection method of ascertainment of cases-controls** | **Same Non response rate in cases and controls** |  |
| 2016 | Ng | 1 | 1 | 1 | 1 | 2 | 1 | 1 | 0 | 8 |
| 2016 | Abu-Amero | 1 | 1 | 0 | 1 | 2 | 1 | 1 | 0 | 7 |
| 2015 | Burdon | 1 | 1 | 1 | 1 | 2 | 1 | 1 | 0 | 8 |
| 2015 | Chen | 1 | 1 | 0 | 1 | 2 | 1 | 1 | 0 | 7 |
| 2015 | Williams | 1 | 1 | 0 | 1 | 2 | 1 | 1 | 0 | 7 |
| 2015 | Philomena-din | 1 | 1 | 0 | 1 | 2 | 1 | 1 | 0 | 7 |
| 2012 | Mabuchi | 1 | 1 | 0 | 1 | 2 | 1 | 1 | 0 | 7 |
| 2014 | Michael | 1 | 1 | 0 | 1 | 2 | 1 | 1 | 0 | 7 |
| 2013 | Liu | 1 | 1 | 1 | 1 | 2 | 1 | 1 | 0 | 8 |
| 2012 | Cao | 1 | 1 | 1 | 1 | 2 | 1 | 1 | 0 | 8 |
| 2012 | Osman | 1 | 1 | 1 | 1 | 2 | 1 | 1 | 0 | 8 |
| 2012 | Dimasi | 1 | 1 | 1 | 1 | 2 | 1 | 1 | 0 | 8 |
| 2012 | Takamoto | 1 | 1 | 0 | 1 | 2 | 1 | 1 | 0 | 7 |
| 2011 | Fan | 1 | 1 | 1 | 1 | 2 | 1 | 1 | 0 | 8 |
| 2011 | Burdon | 1 | 1 | 1 | 1 | 2 | 1 | 1 | 0 | 8 |
| 2014 | Vishal | 1 | 1 | 0 | 1 | 2 | 1 | 1 | 0 | 7 |
| 2017 | Shiga | 1 | 1 | 1 | 1 | 2 | 1 | 1 | 0 | 8 |
| 2017 | Yoshikawa | 1 | 1 | 0 | 1 | 2 | 1 | 1 | 0 | 7 |
| 2019 | Present Study | 1 | 1 | 0 | 1 | 2 | 1 | 1 | 0 | 7 |
